# Cook County Hospital

**Published:** 1874-12

**Authors:** Edwin Powell


### Hospitals.

**Cook County Hospital.** Surgical Clinics. October, 1874. Service of Prof. EDWIN POWELL. Reported by Dr. F. C. WINSLOW, Assistant Physician.

*Popliteal Aneurism, (cured by digital compression in twenty-four hours.)*

CASE 13,644. George M——; Frenchman; aged 38; painter; admitted October 7, 1874. The patient says he always enjoyed excellent health until five months ago, when he began to have a feeling of stiffness in the right leg and knee. While working, he spent a good deal of time standing upon ladders, or was frequently engaged carrying heavy weights up or down. When not busy, it was his custom to throw himself down upon the grass and sleep, thus exposing himself more or less to cold and damp. He continued his work—the stiffness gradually increasing—until about a month ago, when he felt a dull pain in the hollow of the knee. This was accompanied by a swelling a couple of inches in length, and about the size of an earth worm, in the right popliteal space. For the past month the swelling has steadily increased, and the enlargement has been particularly rapid during the past week, the pain, meanwhile, becoming more severe in character. He supposed he had rheumatism, and, in fact, has received liniments, ointments, pills, and kindred medications, at the hand of different doctors both for rheumatism and sprain of the knee.

He is a hearty looking, well nourished man. The appetite lately has been poor; bowels costive; complains of pain in none of the joints except the right knee, and there it is all referred to the popliteal space.

On examination, find an ovoidal tumor larger then a hen's egg, occupying the right popliteal space. It is elastic; pulsates freely in all directions; is smooth and sacculated; the skin glides freely over it, and the superficial veins are enlarged and turgescent. Auscultation reveals a harsh *bruit* all over the tumor, and up the artery as far as Poupart's ligament, while the pulsations of the tumor are entirely controlled by pressure upon the femoral artery.

*Remarks.* After giving a history of the case, the symptoms, diagnosis, prognosis, etc., concerning the treatment, the Prof.

said: It was formerly the practice in treating such tumors, to cut down into the sac, turn out the clots, and ligate the open mouths of the vessel to control the hemorrhage. Such a procedure was almost sure to be followed by a secondary hemorrhage, which proved fatal to the patient. Deligation of the artery on the cardiac side of the tumor generally resulted in a radical cure, but as there was always the possibility of violent inflammation, gangrene, or secondary hemorrhage, it was advisable to try milder means first. He then explained instrumental and digital compression, and the physiological cure effected by these methods. He spoke of forced flexion, and said that in the present case that method would be tried first, and in case it proved unsuccessful, digital compression would next be tried.

October 9. At 4 P. M. the leg was firmly flexed upon the thigh, and confined by a broad strap of adhesive plaster. The thigh was then gently flexed upon the abdomen, and the knee supported by pillows. At 6 P. M. this had become quite painful to him, and morph. sulph. gr. $\frac{1}{8}$ was administered. It was evident that he could not bear the flexion much longer, and accordingly nine men were selected to control the circulation of the femoral artery by making digital compression just below Poupart's ligament, each man to maintain pressure twenty minutes.

At 7 P. M. the compression was begun, the adhesive strap being removed, and the leg extended in an easy position. Pulse, at wrist, 66, and full. At 8 P. M., pulse 68; patient a little restless; morph. gr. $\frac{1}{8}$. At 9 P. M., pulse 72, and full. At 10 P. M. pulse 76; patient easy. At 11 P. M. pulse 76; pressure kept up continuously; he experiences no inconvenience. At 12 midnight, pulse 72, and good; complains a little of cramping; otherwise feels well; leg a little œdematous, and veins somewhat swollen; tumor has a firmer feeling.

October 10. At 12:30 A. M. morph. sulph. gr. $\frac{1}{8}$. At 1 A. M. pulse 72; complains a little of the pressure; tumor feels harder, and pulsations are obscure. There is numbness of the leg, and a decrease of temperature.

At 2 A. M. pulse 72; feels easy. At 3 A. M. pulse 78; comfortble. Sensation is gradually returning from above downwards, and is in exact proportion to the temperature. New vessels can be felt pulsating on the anterior aspect of the knee.

At 4 A. M. pulse 72; still comfortable. A single thickness of soft muslin is interposed between the fingers and the integument. Assistants observe that half the force required at first is now sufficient to control the circulation.

At 5 A. M. pulse 66; feels pretty well. At 6 A. M. pulse 66; patient has slept more or less the last three hours.

At 7 A. M. pulse 72. On removing the pressure only an obscure pulsation is felt at the seat of the tumor. At 10 A. M. pulse 80; very comfortable. At 12 noon, pulse 80; no pain or cramps. Takes beef tea freely. At 4 P. M. pulse 72; nothing can be heard with a stethoscope.

At 7 P. M. there is no pulsation in the tumor, nor does extension of the limb reveal the slightest vibration. Pressure is discontinued twenty-four hours and twenty minutes from its inception.

The patient is in excellent condition; has a little pain in the calf of the leg, and slight tenderness of the integument at Poupart's ligament. The limb is gently flexed, supported by a pillow, and left to itself.

October 11. Feels pretty well; some pain on extending the leg.

October 12. Comfortable.

October 17. Patient up and around the ward.

Thanks are due to Mr. Fredericks and Mr. Mallory, students at Rush Medical College, for assistance rendered in this case.

*Double Fracture of Lower Jaw—Treatment of Chronic Ulcers—Bursa Patellæ—Adulterated Bismuth Subnitras—Necrosis Humerus—Injury to the Skull—Crushing Injury to the Lower Extremities—Retention of Urine.*

I. DOUBLE FRACTURE OF LOWER JAW.—The bone was broken on the right side, just anterior to the angle and on the left side, at a point midway between the angle and the symphysis, the fragment being displaced downwards and backwards. A loop of annealed iron wire was passed around the superior central incisor and the ends twisted together. The corresponding tooth in the lower jaw was treated in a similar manner, and by means of these loops the teeth were approximated, and retained in position by twisting the ends of the wire together. External support was afforded by means of a gutta percha splint, moulded to the chin, and well padded, being confined by several turns of a roller under the jaw and over the vertex.

II. Treatment of Chronic Ulcers.—From half a dozen to a dozen and a half free incisions are made, extending from the edge of the ulcer outwards through the region of deficient circulation, marked by a bluish circle surrounding the ulcer, and down through the skin and cellular tissue. The operation is followed by immediate relief to the congested and constricted vessels, the discolored circle disappears and granulations spring up freely over the indolent surface.

III. Bursa Patellæ.—The bursa was punctured and the liquid evacuated. Pressure was maintained by placing a dry sponge over the bursa, and confining it in its place by a few turns of the roller. Afterward the sponge was moistened, and by its expansion kept up a steady and uniform pressure until the tumor disappeared.

IV. Adulterated Bism. Subnit.—The patient was suffering from an extensive burn of the leg. Bism. subnit. was dusted thickly over the open surface and covered with dry lint. In a few days symptoms of metallic poisoning exhibited themselves; the patient complaining of increased pain at the seat of the of the injury; a metallic taste in the mouth; a blue line being plainly visible upon the gums, and the cheeks were the seat of numerous small ulcers. The bismuth was tested and exhibited the presence of lead in large quantities. The dressing was immediately discontinued, and marked improvement in the condition of the patient followed the substitution of a dressing of simple cerate and the internal administration of potas. iodid. in doses of grs. x. three times daily.

V. Necrosis Humerus—Removal of Diseased Portion—Dressing, etc.—The case is that of a young man; a teamster. The affection came on spontaneously, a year ago. The arm is at present the seat of numerous sinuses leading down to the bone. An opening, six inches in length, was made on the outer aspect of the arm down to the humerus. Here a sort of a pocket was found containing a number of fragments already exfoliated. These were removed and the sides of the cavity scraped clean.

The wound was then packed with cotton, wet in a solution of ac. carbol. For the better cleansing of this cavity, the cotton is removed twice daily, and a stream of carbolated water directed upon the granulating surfaces, through the nozzle of a fountain syringe.

VI. Injury to the Skull—Continuous Cephalalgia—Cured by the Trephine.—The patient is a laborer who, while engaged in chopping down trees, was struck on the head by a falling limb. He was rendered temporarily insensible, but on recovering conciousness exhibited no paralysis, or other evidence of severe lesion. Since this time, however, he has been unable to work on account of constant pain in the head, less severe in the morning, but growing more marked in the evening, and at night, and greatly increased by stooping or lifting.

A well marked depression can be felt at the seat of the injury. A semicircular flap of integument was lifted from the bone, and the trephine applied. The dura mater was found roughened and thickened. No bad symptoms followed the operation, and for three weeks no pain has been experienced.

VII. Crushing Injury of the Lower Extremities—Double Amputation, etc.—The man was brakesman on a freight train, and was brought in with both feet crushed by the wheels of a car. In such cases the injury to the bones and soft parts is usually much greater than the external appearances would indicate, therefore it is always advisable to go at least six inches above the seat of the injury in order to preserve the integrity of the flaps.

Esmarch's bandage was applied, and the circular operation performed. The flaps were only closed at the upper half of the wound, leaving the rest open for free drainage. The stumps were lightly dressed with patent lint, moistened with water slightly carbolated. The subsequent progress of the case has been excellent.

VIII. Retention of Urine without Stricture, Owing to Enlargement of the Middle Lobe of the Prostate Gland.—The patient is an old man who is totally unable to empty the bladder. The ease with which a number twelve sound passes into the bladder, excludes the presence of stricture, while with the finger in the rectum we fail to find sufficient hypertrophy of the entire prostate to cause retention. We therefore conclude that this is a case of retention caused by hypertrophy of the middle lobe of the prostate. This condition manifests itself by the presence of a small conical projection into the bladder, situated just behind the opening into the urethra. This body acts in the same manner as a valve, and by the constantly accumulating urine, is crowded down over the urethra, effectually preventing the emptying of the viscus.

The treatment of such a case consists in carefully instructing the patient to pass a catheter upon himself at regular intervals of six or seven hours. By this means, and by correct hygienic measures, the patient may often pass many years in comparative comfort.

SURGICAL CLINIC. Dr. POWELL. October 13. Reported by RALPH E. STARKWEATHER, M.D. *Popliteal Aneurism of the Right Leg—Treatment by Flexion—Treatment by Digital Compression, and cure in twenty-four hours.*

*Remarks.*—No particulars as to size or location of the tumors were given. The leg had been much swollen and the knee enlarged; the lateral expansion had been considerable; the pain had been constant and severe. The first method employed in this case was by flexion, the limb being strapped. This was continued three hours, morphine being given to lessen pain. The man was unable to endure the treatment. Pressure on the femoral artery by relays of assistants was then ordered, and continued uninterruptedly for twenty-four hours and twenty minutes.

There was not much change in the tumor for the first six hours of pressure; it then began to harden. The cure is complete. There is now no pulsation; the size of the knee is reduced one-third. The tumor is very hard—a mass of consolidated fibrine, which will disappear in from four to six months.

During the past three years I have had seven cases of aneurism, of which five have been treated by digital compression, successfully. It is a method free from danger, and one of comfort and relief to the patient. I have never had a perfect cure when treating by flexion, though in some cases it has been kept up seventy hours. In the treatment by ligature there is danger to a certain extent, and you have a wound which may keep open three or four weeks.

Drawing horse-hairs through the sac, or small, fine wires, is another method of treatment; but in this there is danger of suppuration. If digital pressure were to fail, the galvano-cautery, or injection of ten to thirty drops of the liq. ferri persulphatis, might be effective.

